# *β*-Ga_2_O_3_ Nanostructures: Chemical Vapor Deposition Growth Using Thermally Dewetted Au Nanoparticles as Catalyst and Characterization

**DOI:** 10.3390/nano12152589

**Published:** 2022-07-28

**Authors:** Asha Yadav, Bo Fu, Stephanie Nicole Bonvicini, Linh Quy Ly, Zhitai Jia, Yujun Shi

**Affiliations:** 1 Department of Chemistry, University of Calgary, Calgary, AB T2N 1N4, Canada; ashayadav@eee.sastra.edu (A.Y.); snbonvic@ucalgary.ca (S.N.B.); linh.ly2@ucalgary.ca (L.Q.L.); 2 State Key Laboratory of Crystal Materials, Shandong University, Jinan 250100, China; 201712452@mail.sdu.edu.cn (B.F.); z.jia@sdu.edu.cn (Z.J.)

**Keywords:** gallium oxide *β*-Ga_2_O_3_, semiconductor, nanostructures, nanowires, chemical vapor deposition, photoluminescence

## Abstract

*β*-Ga_2_O_3_ nanostructures, including nanowires (NWs), nanosheets (NSHs), and nanorods (NRs), were synthesized using thermally dewetted Au nanoparticles as catalyst in a chemical vapor deposition process. The morphology of the as-grown *β*-Ga_2_O_3_ nanostructures depends strongly on the growth temperature and time. Successful growth of *β*-Ga_2_O_3_ NWs with lengths of 7–25 μm, NSHs, and NRs was achieved. It has been demonstrated that the vapor–liquid–solid mechanism governs the NW growth, and the vapor–solid mechanism occurs in the growth of NSHs and NRs. The X-ray diffraction analysis showed that the as-grown nanostructures were highly pure single-phase *β*-Ga_2_O_3_. The bandgap of the *β*-Ga_2_O_3_ nanostructures was determined to lie in the range of 4.68–4.74 eV. Characteristic Raman peaks were observed with a small blue and red shift, both of 1–3 cm^−1^, as compared with those from the bulk, indicating the presence of internal strain and defects in the as-grown *β*-Ga_2_O_3_ nanostructures. Strong photoluminescence emission in the UV-blue spectral region was obtained in the *β*-Ga_2_O_3_ nanostructures, regardless of their morphology. The UV (374–377 nm) emission is due to the intrinsic radiative recombination of self-trapped excitons present at the band edge. The strong blue (404–490 nm) emissions, consisting of five bands, are attributed to the presence of the complex defect states in the donor (V_O_) and acceptor (V_Ga_ or V_Ga–O_). These *β*-Ga_2_O_3_ nanostructures are expected to have potential applications in optoelectronic devices such as tunable UV–Vis photodetectors.

## 1. Introduction

One (1D)- and two-dimensional (2D) semiconductor nanostructures such as nanowires, nanorods, nanotubes, nanobelts, and nanosheets have recently attracted extensive research interest due to their unique electrical, optical, and mechanical properties, distinctive from their bulk counterpart. Monoclinic gallium oxide (*β*-Ga_2_O_3_) is a group III metal oxide semiconductor with a wide bandgap of ~4.9 eV and remarkable thermal and chemical stability up to 1400 °C [1,2]. The large surface-area-to-volume ratio and quantum size effect in the 1D and 2D *β*-Ga_2_O_3_ nanostructures have opened up many potential applications in *β*-Ga_2_O_3_ nanostructure-based optoelectronic, electronic, and photonic devices with improved performance [3]. The application of *β*-Ga_2_O_3_ nanostructures in optoelectronic devices is widespread, including solar-blind UV photodetectors [4,5] and gas sensors [6,7]. Furthermore, these nanostructures have also found applications in nanowire-based field-effect transistors [8], nanophotonics such as optical switches [9] and waveguides [10], light-emitting diodes [11], and harsh electronics [12].

*β*-Ga_2_O_3_ has been known to possess luminescence properties due to the presence of oxygen and gallium vacancies [13,14]. Broad emission in the spectral region of UV-blue-green has been observed from bulk *β*-Ga_2_O_3_ upon excitation by UV or cathode ray radiation. The broad UV emission has been attributed to an intrinsic recombination of an electron and a self-trapped hole (STH) [15]. The blue luminescence at 400–490 nm originates from a tunnel recombination of an electron on a donor (an O vacancy) cluster with a hole at an acceptor (a Ga vacancy or a Ga–O vacancy pair) site [13,16]. The green emission, covering a range between 494 nm and 500 nm for the bulk *β*-Ga_2_O_3_, was reported to also be due to the donor–acceptor pair (DAP) recombination [17,18]. The photoluminescent properties of *β*-Ga_2_O_3_ nanowires (NWs) [19,20] and other nanostructures [21,22] have also been reported. The *β*-Ga_2_O_3_ nanostructures also emit UV-blue-green light, similar to the bulk *β*-Ga_2_O_3_. It has been found that the intensities of blue-green emissions depend strongly on the defects formed near the surface region, which is in turn determined by the growth conditions and the structures [23]. In addition, Song et al. showed that nitrogen doping in the *β*-Ga_2_O_3_ NWs can induce an intense red-light emission at around 725 nm (1.71 eV) at room temperature [24]. The red-light emission originates from the recombination of an electron on defect donor states due to O vacancies and a hole trapped on an acceptor due to N doping.

Various methods have been employed for the synthesis of nanosized *β*-Ga_2_O_3_ with various morphologies, structures, and properties. Growth methods such as physical evaporation [25], carbothermal reduction [19,26], DC arc discharge [27,28], laser ablation [29], microwave plasma chemical vapor deposition (MPCVD) [30,31], and thermal CVD [2,8,32,33] have been reported in the literature. Of all these techniques, thermal CVD is regarded as the most commonly used method due to its various advantages, including high efficiency, low production cost, and capability of producing pure materials. In general, two mechanisms, i.e., vapor–liquid–solid (VLS) [23,32,33] and vapor–solid (VS) [25,31,34], govern the vapor-phase growth of *β*-Ga_2_O_3_ nanostructures. In VLS, a metal catalyst is required to form a liquid alloy with Ga_2_O_3_ precipitated at the liquid–solid interface due to supersaturation in the alloy. The VS process is typically catalyst free. The VLS growth leads preferentially to an axial growth, whereas VS gives growth along the radial direction [23,25,35]. Regardless of the various growth techniques and different growth mechanisms, the control of the morphology, the size, and the structure of the as-grown *β*-Ga_2_O_3_ nanostructures is of great importance in determining their properties and applications.

The position and size of the Au NP catalyst play an important role in the final nanostructures in the VLS growth mechanism. In this work, we have used Au nanoparticles (NPs) with uniform size distributions produced by thermal dewetting of thin Au films on Si substrates as catalyst in the CVD growth of *β*-Ga_2_O_3_ nanostructures. The effect of growth parameters, including the temperature and time, on the morphologies of the produced *β*-Ga_2_O_3_ nanostructures, i.e., NWs, nanosheets (NSHs), and nanorods (NRs), was investigated. The growth mechanisms were also discussed. It was found that the morphologies of *β*-Ga_2_O_3_ nanostructures could be controlled by the growth parameters. From it, the conditions leading to predominant NW growth via the VLS mechanism were identified. In addition, the structural and optical properties of the synthesized *β*-Ga_2_O_3_ nanostructures were studied using X-ray diffraction (XRD), Raman spectroscopy, UV–Vis diffuse reflectance spectroscopy, and photoluminescence (PL) spectroscopy.

## 2. Experimental Methods

Ga_2_O_3_ nanostructures were grown by the CVD method using Au NPs as catalyst. The Au NPs were produced using the technique of thermal dewetting of thin Au metallic films. For this, Au thin films with a 2.9 nm thickness were sputter coated on Si (100) substrates using a sputter deposition system (BAL-TEC SCD 500) at a constant current of 40 mA under high-purity Ar (99.998%, Praxair, Calgary, AB, Canada) at a pressure of 10 mTorr. An internal Bal-TEC QSG 100 quartz crystal film thickness monitor was used to measure the actual film thickness during sputtering. Before sputtering, the phosphorous-doped *n*-type Si (100) wafers with a resistivity of 1–20 Ω cm (Wafer World Inc., West Palm Beach, FL, USA) were cleaned successively with acetone, ethanol, and methanol, and dried using high-purity N_2_ gas (99.999%, Praxair). Details of thermal dewetting to make metal NPs were described previously [36,37,38]. Briefly, thermal dewetting of 2.9 nm thick Au films on Si substrates was carried out in vacuum (~2 × 10^−3^ Torr) at 600 °C for 1 h.

The growth of Ga_2_O_3_ nanostructures was carried out in a quartz tube with an inner diameter of 46 mm that was enclosed in a conventional horizontal tubular furnace (Lindberg Blue M, Thermo Scientific, Waltham, MA, USA). About 0.5 g of Ga metal (99.999%, Aladdin) was placed in a quartz boat, and the Au NP-coated Si substrates were placed in the same boat at a distance of 2 cm downstream. The quartz boat was then loaded into the center of the quartz tube. The tube was heated to the desired growth temperature in the range of 800–1100 °C at a constant flow of 50 sccm Ar gas (99.999%, Praxair) maintained during the growth process at atmospheric pressure. The growth time was varied from 25 to 60 min. After completion of the deposition, the furnace was allowed to cool down to room temperature under constant Ar flow.

The morphology of the as-grown Ga_2_O_3_ nanostructures was characterized using a field-emission scanning electron microscope (FESEM, Zeiss Sigma VP, Oberkochen, Germany) under ultra-high vacuum (<10^−10^ Torr) at an accelerating voltage of 10 kV. Energy dispersive X-ray (EDX) spectroscopic analysis was performed using an INCA x-act EDX system (Oxford Instruments, Abingdon, UK). The diameter and length of the produced NWs were measured using the ImageJ software. The XRD patterns were collected for a 2θ range of 10–80° at a scan rate of 0.04° (2θ)/s using a Bruker D8 Advance X-ray diffractometer with Cu Kα radiation (λ = 1.54178 Å). The Raman spectra were recorded on a Raman spectrometer (Horiba XploRA Plus) with a laser wavelength of 532 nm from a diode-pumped solid-state laser and a Syncerity OE detector. All spectra were recorded at room temperature at a laser power of 14 mW with a 1 cm^−1^ resolution and an error of ± 0.5 cm^−1^ using a 10× objective in the scan range of 100 to 800 cm^−1^. The PL spectra were obtained at room temperature using a fluorescence spectrophotometer (Horiba FluoroMax-4) with a Xe lamp as an excitation source and a solid sample holder. The bandgap of *β*-Ga_2_O_3_ nanostructures was determined from the UV–Vis diffuse reflectance spectra measured using a UV–Vis–NIR spectrophotometer (Cary 5000) equipped with a DRA-2500 diffuse reflectance accessory with an integrating sphere in the scan range of 200–800 nm.

## 3. Results and Discussion

### 3.1. Morphological and Structural Characterization and Growth Mechanism

**Figure 1:**
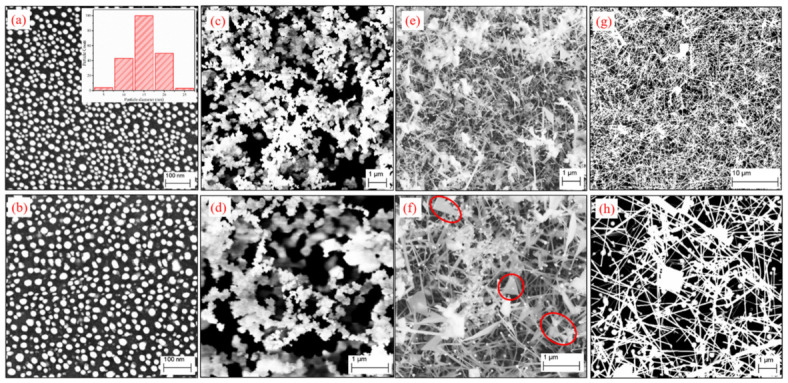
FESEM images of (**a**) Au nanoparticles after thermal dewetting of a 2.9 nm thick Au film at 600 °C for 1 h. The inset shows the size distribution of the produced NPs. FESEM images of the as-grown Ga_2_O_3_ nanostructures prepared at (**b**) 800 °C, (**c**,**d**) 900 °C, (**e**,**f**) 1000 °C, and (**g**,**h**) 1100 °C for a growth time of 32 min under a constant Ar flow of 50 sccm. (**c**,**d**), (**e**,**f**), and (**g**,**h**) are for the same growth temperature but at different magnification in FESEM.

Au NPs were produced on Si substrates by the technique of thermal dewetting and employed as catalyst in the CVD growth of Ga_2_O_3_ nanostructures. Figure 1a shows the FESEM image of the Au NPs after thermal dewetting of a 2.9 nm thick Au film on a Si substrate at 600 °C for 1 h, with the size distributions of the Au NPs shown in the inset. The produced Au NPs are uniformly distributed on the substrate with an average diameter of 18 ± 4 nm.

The FESEM images of Au-catalyzed Ga_2_O_3_ nanostructures are shown in Figure 1b–h for samples grown at different growth temperatures (T) between 800 and 1100 °C under a constant Ar flow of 50 sccm at a growth time of 32 min and a Ga-to-substrate distance of 2 cm. At T = 800 °C, no growth of Ga_2_O_3_ NWs or NSHs was observed (Figure 1b); however the size of the NPs showed an increase to 22 ± 5 nm. The same was observed for T = 850 °C in that there was no growth of Ga_2_O_3_ NWs or NSHs either, and the NP size further increased to 25 ± 7 nm. Auer et al. also observed an increase in the NP diameter before the growth of Ga_2_O_3_ nanostructures at the low temperature of 800 °C when using Au NPs as catalyst, and they ascribed it to the condensation of Ga from the vapor phase leading to a swelling of the NPs [32]. At growth temperatures of 900 and 950 °C, the morphology of the as-grown samples showed poorly defined nanoclusters, with the representative FESEM images for 900 °C at low and high magnification presented in Figure 1c,d, respectively. As the temperature increased to 1000 °C, Ga_2_O_3_ NWs started to form along with quite a lot of triangular-shaped NSHs (Figure 1e,f). The average diameter of the NWs prepared at 1000 °C is 47 ± 14 nm, and the average length of these NWs is 8 ± 2 μm. With a further increase in temperature to 1100 °C, the morphology is now predominantly NWs with much fewer NSHs (Figure 1g,h). The average diameter and length of the NWs grown at 1100 °C are 76 ± 23 nm and 16 ± 3 μm, respectively. The produced Ga_2_O_3_ NWs have good diameter distributions thanks to the use of uniformly distributed Au nanoparticles as catalyst, illustrating the important role that the size of the Au catalyst plays on the size of the produced NWs. Compared to the samples produced at 1000 °C, both the diameter and length of the NWs increase with the growth temperature. The higher Ga vapor pressure at increased temperatures is believed to be responsible for the growth of longer NWs at higher temperatures, and the induced supersaturation in catalyst alloys may have resulted in the larger diameters in the NWs. The growth rate of Ga_2_O_3_ NWs of 0.25 and 0.50 μm min^−1^ at 1000 and 1100 °C, respectively, is higher than the ones at 0.052–0.075 μm min^−1^ reported under similar conditions, but at a lower T of 900 °C [39]. It should also be noted that no Ga_2_O_3_ nanostructures were observed in our experiments when using blank Si substrate without the Au NPs, indicating that Au NPs are needed for the growth of Ga_2_O_3_ nanostructures.

**Figure 2:**
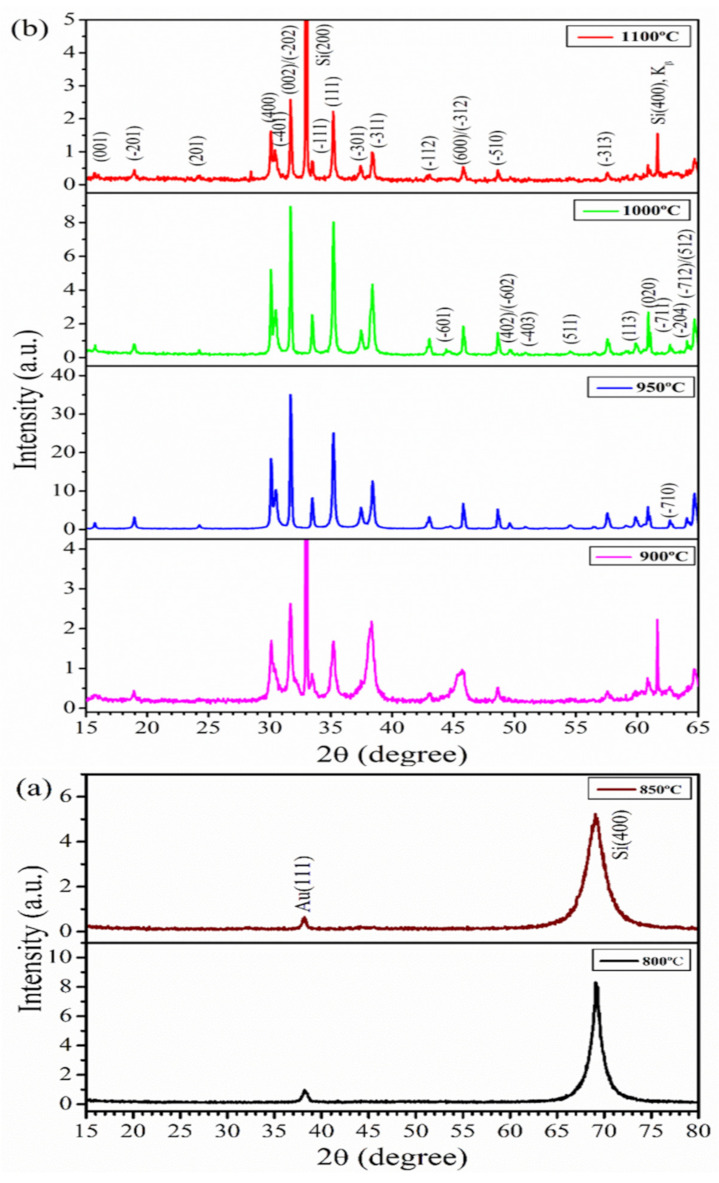
XRD patterns of *β*-Ga_2_O_3_ nanostructures grown at different temperatures of (**a**) 800 and 850 °C in the 2θ range of 15–80°, and (**b**) 900–1100 °C in the 2θ range of 15–65° under a constant Ar flow of 50 sccm for 32 min.

The structures and phase purity of the as-grown Ga_2_O_3_ nanostructures were characterized by XRD. Figure 2 shows the XRD patterns collected from the samples grown at different temperatures of 800–1100 °C. At low temperatures of 800 and 850 °C, no diffraction peaks of *β*-Ga_2_O_3_ were found, as shown in Figure 2a. The peaks from Au(111) and Si(400) were present, which is similar to those from the Au-NP-coated Si substrate, as shown in Figure S1a in the Supplementary Materials. This agrees with the FESEM results in Figure 1b, which showed no growth of *β*-Ga_2_O_3_ nanostructures at 800 °C. As the temperature increased to 900 °C, the Au(111) and Si(400) peaks were still present along with other weak peaks from the Si substrates (i.e., Si(200), and Si(400) from Cu Kβ radiation—see Figure S1b in the Supplementary Materials). However, new XRD peaks other than the above were observed, as indicated by the XRD patterns shown in Figure 2b for this temperature and other temperatures at 950–1100 °C. For these XRD spectra in Figure 2b, only 2θ values up to 65 °C were shown to avoid the overloading of the Si (400) peak. These new XRD peaks can all be indexed to the *β*-phase of Ga_2_O_3_ with a monoclinic structure according to ICDD 41-1103, with the strongest peaks assigned to (400), (002)/(-202), (111), and (-311). With a further increase in temperature to 950 and 1000 °C, the intensities of all *β*-Ga_2_O_3_ peaks increased with a total of 24 peaks at 1000 °C. No other phases (α, γ, δ, and ε) of Ga_2_O_3_ were observed in the XRD analysis of our as-grown samples, indicating that high-purity and single-phase *β*-Ga_2_O_3_ nanostructures were produced from the CVD growth. As the density of NWs decreased for the sample prepared at T = 1100 °C as observed in the FESEM image (Figure 1h), the diffraction peak intensities of *β*-Ga_2_O_3_ were reduced, accompanied by an increase in the peaks from the Si substrate (i.e., (200) and (400)).

**Figure 3:**
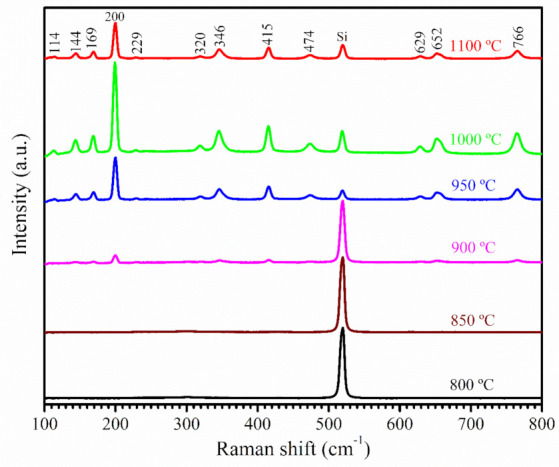
Room-temperature Raman spectra of *β*-Ga_2_O_3_ NWs prepared at various temperatures from 800 to 1100 °C for 32 min.

Figure 3 shows the room-temperature Raman spectra of the *β*-Ga_2_O_3_ nanostructures prepared at different growth temperatures in the frequency range of 100–800 cm^−1^. The Raman spectra of the samples at 800 and 850 °C only showed the peak at 520 cm^−1^ from the crystalline Si substrate, in excellent agreement with the results from XRD and FESEM analysis indicating no growth of *β*-Ga_2_O_3_ nanostructures at these two temperatures. Eleven out of the fifteen Raman active modes as reported in the literature for bulk *β*-Ga_2_O_3_ [40] were observed for samples prepared at temperatures >900 °C. The sharp peaks from *β*-Ga_2_O_3_ in the Raman spectra as shown in Figure 3 indicate good crystallinity in the prepared samples, which agrees well with the XRD analysis of the same samples in Figure 2. Dohy et al. classified the 15 Raman active modes of bulk monoclinic *β*-Ga_2_O_3_, belonging to the C_2h_ group, into three regions [40]. They are the low-frequency (≤200 cm^−1^), mid-frequency (300–500 cm^−1^), and high-frequency (500–800 cm^−1^) modes, related to the libration and transformation of the Ga_2_O_6_ octahedra—GaO_4_ tetrahedra chain, deformation of the Ga_2_O_6_ octahedron, and stretching and bending of the GaO_4_ tetrahedron, respectively. If we compare the Raman peaks of the *β*-Ga_2_O_3_ NWs and NSHs produced at 1000 and 1100 °C with those from the bulk material, a very small red shift of 1–3 cm^−1^ was observed mostly in the low-frequency region, whereas a very small blue shift of 1–3 cm^−1^ appeared towards the high-frequency region. Gao et al. [26] reported a red shift of 4–23 cm^−1^ for the *β*-Ga_2_O_3_ nanorods, and Dai et al. [21] found a 30 cm^−1^ red shift for the nanobelts and NSHs as compared to the bulk *β*-Ga_2_O_3_ powder. The red shift was attributed to the presence of defects and the oxygen vacancies in the nanostructures. On the other hand, Rao et al. [41] and Hosein et al. [34] observed a blue shift of 10–40 cm^−1^ and ca. 5 cm^−1^, respectively, in their *β*-Ga_2_O_3_ NWs relative to the bulk. It was argued that the internal strain in the NWs led to the blue shift in the Raman peaks. The observed small red and blue shifts in this work indicated the presence of defects and strains in the *β*-Ga_2_O_3_ NWs and NSHs produced by the CVD method, but the extent was relatively low compared to the *β*-Ga_2_O_3_ nanostructures previously reported [21,26,41].

**Figure 4:**
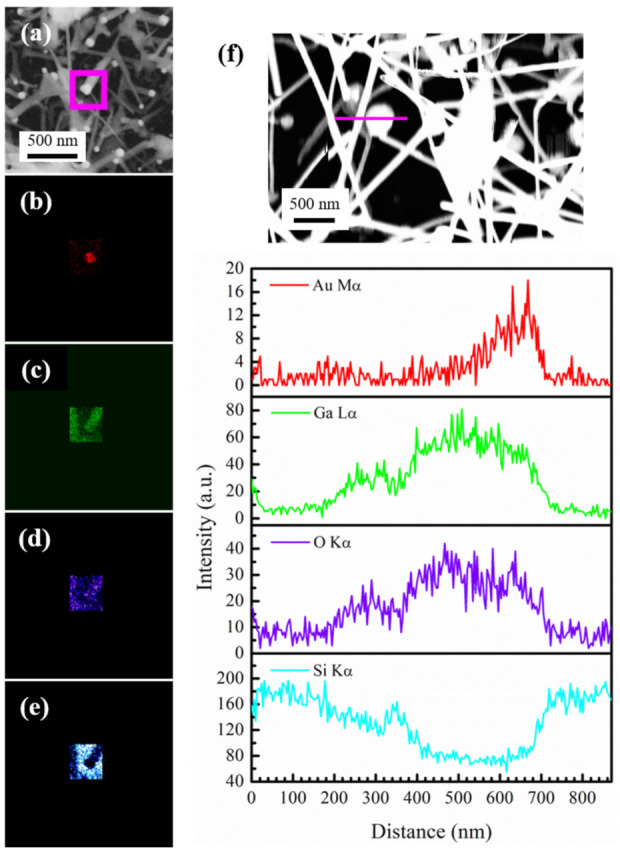
(**a**) FESEM image of *β*-Ga_2_O_3_ NWs grown at 1000 °C and EDX elemental mapping for (**b**) Au (red, Mα), (**c**) Ga (green, Lα), (**d**) O (purple, Kα), (**e**) Si (cyan, Kα) over the area marked by the square box in magenta in (**a**), and (**f**) EDX line scan across a nanoparticle on the tip of a nanowire as indicated by the magenta line in the FESEM image of *β*-Ga_2_O_3_ NWs grown at 1100 °C.

As indicated in Figure 1, *β*-Ga_2_O_3_ NW growth was achieved at temperatures of 1000 and 1100 °C. From Figure 1e,f,h, nanoparticles can clearly be seen at the tip of the NWs. To investigate the composition of the NPs at the NW tips, EDX elemental mapping was performed on an area containing the NPs on the *β*-Ga_2_O_3_ NWs grown at 1000 °C, which is shown in Figure 4a–e for Au (Mα, red), Ga (Lα, green), O (Kα, purple), and Si (Kα, cyan). The EDX mapping clearly shows that the terminal NP is made of Au and the NW stem is made of Ga and O. Figure 4f illustrates an EDX line scan across a nanoparticle on the tip of a *β*-Ga_2_O_3_ NW prepared at 1100 °C, which also shows the presence of Au in the NPs. The unambiguous evidence of the presence of Au catalyst NPs at the top of the *β*-Ga_2_O_3_ NWs demonstrates that the formation of *β*-Ga_2_O_3_ NWs follows the VLS mechanism, in good agreement with other reports in the literature [23,32]. At 1000 °C, aside from the *β*-Ga_2_O_3_ NWs, triangular-shaped NSHs were also observed, as represented in those marked in red circles in Figure 1f. Similar kinds of triangular-shaped nanostructures were reported in previous work on the CVD growth of *β*-Ga_2_O_3_ NWs [2,33,42], and they were described as triple junction nodes and termed as nanoflags. The growth of 2D sheet-like nanoflag structures was ascribed to the nucleation sites formed on the surface of NWs, following a VS mechanism. The VS mechanism leading to the formation of other 2D NSHs than nanoflags was also found in other studies [32,43,44]. Therefore, the nanoflags produced in our work are believed to be due to the VS mechanism from the secondary nucleation sites established on the NW surface by the continuously supplied Ga vapor. Du et al. argued that when the supersaturated vapor pressure in the NWs increased to higher than the equilibrium vapor pressure of the cylindrical sidewall of NWs, growth in the radial direction driven by a VS mechanism started [35]. This explains why the nanoflags are typically grown in the direction perpendicular to the axial direction of NWs in our work. Therefore, two types of growth mechanism, i.e., VLS and VS, proceeded in the formation process of *β*-Ga_2_O_3_ nanostructures via CVD. The Au-catalyzed growth of *β*-Ga_2_O_3_ NW is due to the VLS mechanism, and the VS mechanism is responsible for the radial growth, leading to 2D sheet-like nanoflags. At the growth temperature of 1100 °C, a combination of VLS and VS still operates. However, there are much fewer 2D NSHs as compared to those at 1000 °C, suggesting a dominant VLS growth of *β*-Ga_2_O_3_ NWs at this growth temperature.

**Figure 5:**
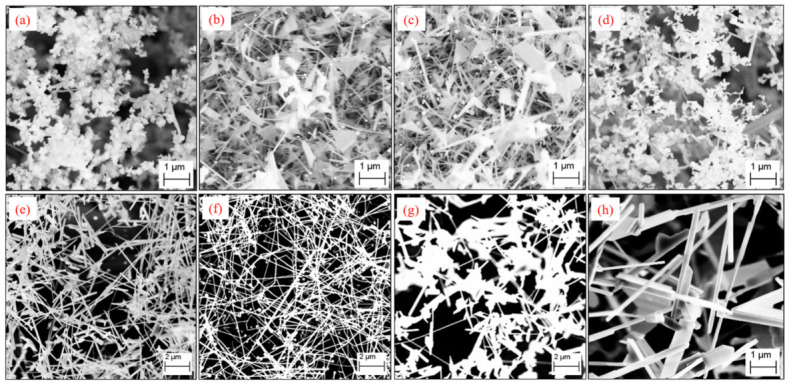
FESEM image of the as-grown *β*-Ga_2_O_3_ nanostructures prepared at 1000 °C for different growth time of (**a**) 30 min, (**b**) 31 min, (**c**) 34 min, (**d**) 35 min, and at 1100 °C for different growth time of (**e**) 30 min, (**f**) 35 min, (**g**) 45 min, and (**h**) 60 min under a constant Ar flow of 50 sccm.

In order to understand the effect of growth parameters on the competition of the VLS and VS mechanisms, we further investigated the growth of *β*-Ga_2_O_3_ nanostructures for different growth times at the two temperatures of 1000 and 1100 °C. Figure 5 shows the FESEM images of the as-grown *β*-Ga_2_O_3_ nanostructures at deposition times varied from 30 to 35 min for 1000 °C (Figure 5a–d) and from 30 to 60 min for 1100 °C (Figure 5e–h) under a constant Ar flow of 50 sccm at a Ga-to-substrate distance of 2 cm. The XRD patterns for the nanostructures prepared for different growth times at the temperatures of 1000 °C and 1100 °C are shown in Figure S2a,b, respectively. All the diffraction peaks corresponded to the monoclinic *β*-Ga_2_O_3_ phase, demonstrating the formation of pure *β*-Ga_2_O_3_ nanostructures. Furthermore, characteristic Raman peaks of *β*-Ga_2_O_3_ were observed for the nanostructures grown at different times for the two temperatures, as shown in Figure S3a,b. Similar to the earlier temperature series, the Raman peaks for these nanostructures showed a small red shift and a small blue shift, both of 1–3 cm^−1^, as compared to the bulk *β*-Ga_2_O_3_.

For the growth time of 30 min (Figure 5a) and 35 min (Figure 5d) at 1000 °C, the as-grown nanostructures are the poorly defined nanoclusters, similar to those observed for samples grown at the lower temperatures of 900–950 °C and 32 min (Figure 1c,d). The growth of nanoclusters was also observed at 25 min at this temperature of 1000 °C. Interestingly, with a small change in the growth time from 30 min to 31 and 34 min, NWs, nanoflags, and nanosheets of other shapes (e.g., rectangular) were observed, which is similar to the morphology of the sample prepared at 32 min at the same temperature of 1000 °C. In addition, the Au NPs were observed on top of most NWs in the samples prepared at 31 and 34 min, suggesting a VLS mechanism for the NW growth. The co-presence of NWs and NSHs at 31 and 34 min indicated that a combination of VLS and VS mechanisms operated under these conditions. The diameters of the *β*-Ga_2_O_3_ NWs grown at 31 and 34 min were determined to be 57 ± 20 nm and 48 ± 18 nm, respectively, with the corresponding NW length of 7 ± 2 μm and 9 ± 3 μm. Compared to the NW diameter and length values, respectively, at 47 ± 14 nm and 8 ± 2 μm for the sample prepared at 32 min, there is not much change in the dimensions of the *β*-Ga_2_O_3_ NWs with the change in deposition time at 1000 °C. This is most likely due to the short time intervals. The deposition time longer than 35 min for 1000 °C was not explored as 35 min already led to the growth of poorly defined nanoclusters.

At an increased temperature of 1100 °C, previous deposition at 32 min led to a predominant growth of *β*-Ga_2_O_3_ NWs (Figure 1g,h) via the VLS mechanism. A slightly shorter deposition time of 30 min still provided predominantly NWs with a small amount of NSHs, as shown in Figure 5e. This is similar to the 32 min deposition, showing a dominant VLS with a minor contribution from VS deposition at 35 min leads to an almost perfect growth of NWs with very little NSHs. This is the best condition for *β*-Ga_2_O_3_ NW growth of all that has been studied in this work. It was found that the *β*-Ga_2_O_3_ NWs grown at 35 min had similar diameters (73 ± 27 nm) to those at 32 min (76 ± 23 nm); however, the *β*-Ga_2_O_3_ NWs grown at 30 min had much larger diameters (184 ± 60 nm) than those of 32 and 35 min at the same temperature of 1100 °C. The length of the *β*-Ga_2_O_3_ NW synthesized at 1100 °C increased with increasing growth time, changing from 11 ± 2 μm at 30 min, to 16 ± 3 μm at 32 min, and finally to 25 ± 8 μm at 35 min. It should be noted that the length of the *β*-Ga_2_O_3_ NWs more than doubled when the growth time changed from 30 to 35 min. The growth rate also increased from 0.37 μm min^−1^ to 0.71 μm min^−1^ within the short period of 5 min. When the growth time was increased to 45 min, the number of *β*-Ga_2_O_3_ NWs decreased significantly (Figure 5g). Instead, the morphology showed a dominant appearance of NSHs along with some short NRs. With a further increase in the deposition time to 60 min, the samples consisted mainly of NRs and thicker NSHs (Figure 5h). Venithakumari et al. synthesized *β*-Ga_2_O_3_ NRs by heating Ga at a high temperature in ambient air without any catalyst and proposed a VS mechanism for the nanorod growth [11]. Auer et al. used the CVD method, similar to ours, with Au catalysts for the growth of *β*-Ga_2_O_3_ NWs, NRs, and nanoribbons, and they ascribed the growth of NRs and nanoribbons to the VS mechanism [32]. Due to the absence of NP catalysts atop the NRs, the NRs observed in our work are supposed to also be from the VS mechanism. Therefore, at the growth temperature of 1100 °C, as the growth time increases from 35 min to 60 min, the growth is switched from a predominant VLS to VS mechanism, strongly suggesting that the growth time plays a significant role in the governing mechanism for the growth of *β*-Ga_2_O_3_ nanostructures.

### 3.2. Bandgap Analysis

**Figure 6:**
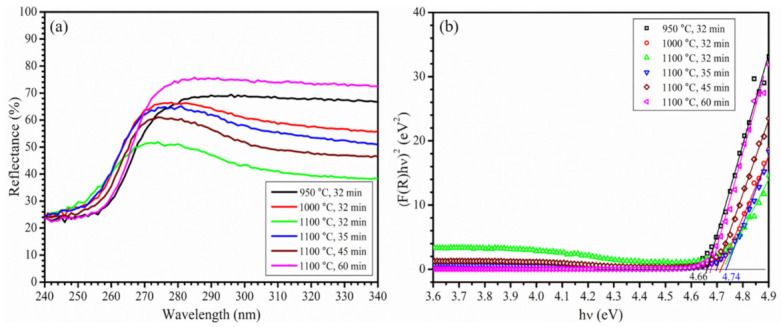
(**a**) Diffuse reflectance spectra, and (**b**) a plot of (*F*(*R*)·h*ν*)^2^ as a function of photon energy for the determination of bandgap of as-grown *β*-Ga_2_O_3_ nanostructures prepared at 950 °C and 32 min (black), 1000 °C and 32 min (red), 1100 °C and 32 min (green), 1100 °C and 35 min (blue), 1100 °C and 45 min (wine), and 1100 °C and 60 min (magenta).

The bandgaps of the as-prepared *β*-Ga_2_O_3_ nanostructures were determined from the diffuse reflectance spectra acquired using the UV–Vis spectrometer. Figure 6a shows the UV–Vis reflectance spectra for the six samples prepared at 950–1100 °C for 32 min and 1100 °C for 35–60 min. The measured reflectance was then transformed to the Kubelka–Munk function, *F*(*R*), using the Kubelka–Munk equation [45]. The *F*(*R*) is related to the bandgap, *E_g_*, according to the following Equation (1):

(1) $${\left(F\left(R\right)\cdot h\nu \right)}^{1/\gamma }=B\left(h\nu -E_{g}\right)$$

where h is the Planck constant, *ν* is the photon frequency, and B is a constant. *γ* is ½ and 2 for direct and indirect bandgap semiconductors, respectively. Since *β*-Ga_2_O_3_ has a direct bandgap, *γ* equals to ½. The plots of (*F*(*R*)·h*ν*)^2^ as a function of photon energy (h*ν*) for the six samples are shown in Figure 6b.

The bandgap for the sample grown at 1100 °C for 35 min, which consists predominantly of *β*-Ga_2_O_3_ NWs, was determined to be 4.74 eV, whereas the value for the *β*-Ga_2_O_3_ nanoclusters grown at 950 °C for 32 min was 4.66 eV. Although the two values are similar, the one for the *β*-Ga_2_O_3_ NWs is slightly higher than that for the nanoclusters. The two other samples prepared at 1100 °C and 1000 °C for a growth time of 32 min, consisting of *β*-Ga_2_O_3_ NWs and NSHs, had their bandgap determined to be 4.73 eV and 4.72 eV, respectively, which is similar to the bandgap of *β*-Ga_2_O_3_ NWs. The two samples that were predominantly NSHs and NRs grown at 1100 °C for 45 and 60 min showed a slightly lower bandgap at 4.70 eV and 4.68 eV, respectively. The bandgap for the *β*-Ga_2_O_3_ NWs and NSHs at 4.72–4.74 eV is in excellent agreement with the values (4.70–4.80 eV) reported for *β*-Ga_2_O_3_ NWs and nanoribbons [46,47]. These bandgap values are slightly smaller than the one at 4.9 eV for bulk crystals. This is possibly due to the defects in these nanostructures [47], consistent with the analysis by the Raman spectra.

### 3.3. Photoluminescence Properties

**Figure 7:**
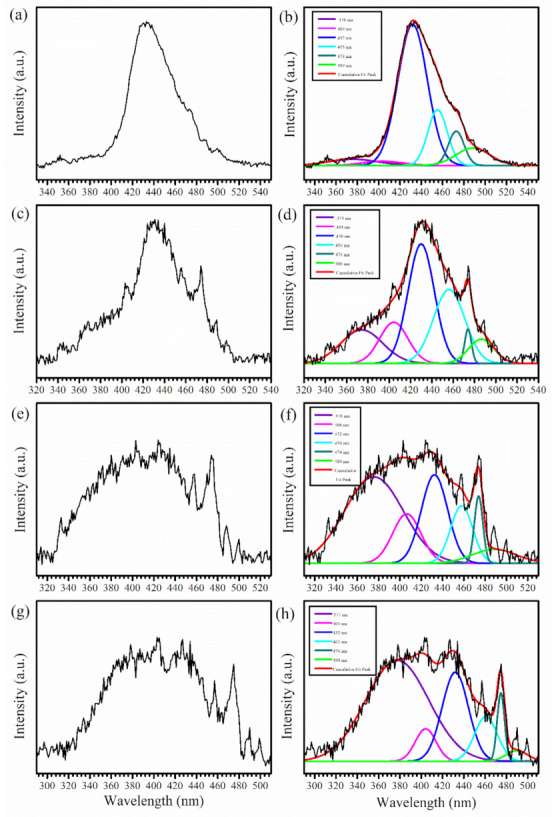
PL spectra of the *β*-Ga_2_O_3_ nanostructures prepared at 1000 °C and 32 min at different λ_ex_ of (**a**) 310 nm, (**c**) 300 nm, (**e**) 290 nm, (**g**) 270 nm, and the corresponding deconvoluted spectra showing emission bands in the UV (purple) and blue (404–406 nm: magenta; 430–432 nm: blue; 455–461 nm: cyan; 473–474 nm: dark cyan; 486–490 nm: green) regions at different λ_ex_ of (**b**) 310 nm, (**d**) 300 nm, (**f**) 290 nm, and (**h**) 270 nm.

The photoluminescence (PL) properties of the synthesized *β*-Ga_2_O_3_ nanostructures were studied. Broad PL emission in the UV-blue spectral region was observed in the samples grown at temperatures ≥900 °C and 32 min. No PL signals were observed from samples prepared at 800 and 850 °C since there were no *β*-Ga_2_O_3_ nanostructures formed at the two lower temperatures. Figure 7 shows the representative PL spectra in the spectral range of 290–550 nm obtained for the *β*-Ga_2_O_3_ nanostructures prepared at 1000 °C and 32 min at different excitation wavelengths. The PL spectrum at an excitation wavelength (λ_ex_) of 310 nm (Figure 7a) showed a broad peak centered around 433 nm. In order to have a clear understanding about the observed luminescence, the PL spectrum was deconvoluted. The deconvolution of this PL spectrum, illustrated in Figure 7b, showed six emission bands with each Gaussian curve centered at 376, 404, 432, 455, 473, and 489 nm, of which the UV (376 nm) and deep blue (404 nm) bands are broad and very weak. To better understand the observed PL spectra in these *β*-Ga_2_O_3_ nanostructures, the sample was excited with different excitation wavelengths (λ_ex_) varied from 310 to 260 nm, with those at 300 nm, 290 nm, and 270 nm shown in Figure 7c,e,g, respectively. The corresponding deconvoluted spectra are illustrated in Figure 7d,f,h, respectively. The PL spectrum at λ_ex_ = 300 nm had similar emission peaks at 374, 404, 430, 456, 474, and 486 nm. With the reduction in λ_ex_ to 290 and 270 nm, the five blue emission bands (404–490 nm) and the UV emission were consistently present, but the UV emission peak became quite dominant at the two shorter λ_ex_ of 290 and 270 nm.

The origin of the PL emission peaks in bulk [13,14,15,16,17] and nanostructured *β*-Ga_2_O_3_ [2,3,20,21,22,23,24] has been extensively studied in the literature. It has been suggested that the UV emission is due to the presence of STHs near the valence band edge and its recombination with electrons. The blue emissions originate from the radiative recombination of donor (an O vacancy—V_O_) and acceptor (a Ga–O vacancy pair—V_Ga_–V_O_ or a Ga vacancy—V_Ga_) pair (DAP), which exist deep in the bandgap. In bulk *β*-Ga_2_O_3_ crystals, the blue emissions consist of three bands due to the different crystallographic sites for Ga and O in the crystal structure of *β*-Ga_2_O_3_ [14,17]. In our work, the intrinsic UV emission (374–377 nm) and five blue emission bands at 404–406 nm (deep blue), 430–432, 455–461, 473–474, and 486–490 nm were observed for the *β*-Ga_2_O_3_ NW and NSH samples prepared at 1000 °C and 32 min, of which the emission at 430–432 nm was the strongest, followed by those at 455–461 nm and 473–474 nm. All other samples prepared at different times at 1000 °C and 1100 °C show similarly strong PL emissions in the UV-blue spectral regions. With the variation in excitation wavelength, it was clearly found that at lower λ_ex_ (i.e., higher excitation energy) closer to the bandgap, the UV emission due to the radiative recombination of self-trapped excitons present at the band edge was dominant. The strong blue emission is due to the abundance of deep donor and acceptor states that naturally existed inside the nanostructures such as NWs and NSHs of *β*-Ga_2_O_3_ [21,22,24,48]. Chang et al. also observed multiple blue emission bands from their *β*-Ga_2_O_3_ NW samples grown on Si or sapphire substrates [49]. In the study of the temperature-dependent PL spectra of *β*-Ga_2_O_3_ nanostrips and their band structure, Ho et al. ascribed the different blue emission bands to the different acceptor states formed by V_Ga_ or the V_Ga_–V_O_ pair [22]. Our observed blue emission bands were in excellent agreement with the various blue emission bands reported in the work of Ho et al. [22], which were assigned to the luminescence from the donor band to the quantum-well states for acceptor clusters by the V_Ga_–V_O_ pair. Therefore, the PL spectra reported in this work provide experimental support for the existence of the quantum-well states in the acceptors formed by the V_Ga_–V_O_ pair in the synthesized *β*-Ga_2_O_3_ nanostructures. The fact that the blue emissions increase in intensity as λ_ex_ increases, i.e., at lower excitation energy or excitation within deep bandgap states, strongly indicates the presence of the donor (V_O_) and acceptor (V_Ga_ or V_Ga–O_) pairs deep inside the bandgap. Villora et al. reported that these O and Ga vacancies were generated at higher growth temperatures [14]. The growth temperatures in our work were high as well (≥900 °C), which also demonstrated that there would be sufficient O and Ga vacancies present in these as-grown NWs and NSHs, leading to the observed strong blue emissions.

## 4. Conclusions

The uniformly distributed Au NPs produced from thermal dewetting of Au thin films were used as catalyst in the CVD growth of *β*-Ga_2_O_3_ nanostructures. The effect of growth temperature and time on the morphology of *β*-Ga_2_O_3_ nanostructures was explored. No growth of *β*-Ga_2_O_3_ nanostructures was found at temperatures lower than 900 °C. The *β*-Ga_2_O_3_ nanocluster, NWs, NSHs and NRs were synthesized depending on growth temperatures and time. CVD growth at 1000 °C and 35 min reproducibly led to a predominant growth of NWs with lengths of 25 ± 8 μm. Deposition at 1000 °C for 31–34 min and at 1100 °C for 30–32 min produced shorter NWs (7–9 μm at 1000 °C and 11–16 μm at 1100 °C) along with the presence of NSHs, whereas the preparation at 1100 °C and 60 min gave NRs. Due to the use of uniformly distributed Au NPs as catalyst, the produced *β*-Ga_2_O_3_ NWs showed good diameter distributions. It has been concluded that the NW growth follows the VLS mechanism, and the growth of NSHs (nanoflags and other shapes) and NRs follows the VS mechanism. Structural analysis from XRD revealed that the as-grown nanostructures are made of highly pure single-phase *β*-Ga_2_O_3_. Regardless of the morphology, the bandgap of the synthesized *β*-Ga_2_O_3_ nanostructures falls within the range of 4.68–4.74 eV. The Raman spectra acquired from these nanostructures exhibited characteristic peaks of *β*-Ga_2_O_3_, in the low-, mid-, and high-frequency regions. A comparison of the Raman peak of these *β*-Ga_2_O_3_ nanostructures with those of the bulk showed a very small blue shift of 1–3 cm^−1^, originating from the internal strain, and a very small red shift of 1–3 cm^−1^, indicating the presence of defects in the as-grown *β*-Ga_2_O_3_ nanostructures. The *β*-Ga_2_O_3_ NWs, NSHs, NRs, and nanoclusters all showed strong PL emission in the UV-blue spectral region. The dominance of the UV emission (374–377 nm) at lower λ_ex_ or higher excitation energy closer to the bandgap is in good agreement with its origin from the radiative recombination of self-trapped excitons present at the band edge. The presence of the complex defect states in the donor (V_O_) and acceptor (V_Ga_ or V_Ga–O_) has led to strong blue emissions consisting of five bands centered at 404–406 nm (deep blue), 430–432, 455–461, 473–474, and 486–490 nm in the synthesized *β*-Ga_2_O_3_ nanostructures. The controlled growth of different morphologies of *β*-Ga_2_O_3_ and the strong PL emissions from these nanostructures will open up opportunities for their application in various optoelectronic devices such as tunable UV–Vis photodetectors.

## Data Availability

All data supporting reported results are included within the article including the supplementary materials.

## Supplementary Materials

The following supporting information can be downloaded at: https://www.mdpi.com/article/10.3390/nano12152589/s1, Figure S1: XRD patterns of (**a**) Au nanoparticles from thermal dewetting of a 2.9 nm thick Au thin film on a Si substrate, and (**b**) a blank Si (100) substrate. Peak assignments are based on ICDD No. 04-0784 for Au and No. 80-0018 for Si; Figure S2: XRD patterns of *β*-Ga_2_O_3_ nanostructures grown for various times of (**a**) 31–34 min at 1000 °C and (**b**) 30–60 min at 1100 °C under a constant Ar flow of 50 sccm; Figure S3: Room-temperature Raman spectra of *β*-Ga_2_O_3_ nanostructures grown for various times of (**a**) 31–34 min at 1000 °C and (**b**) 30–60 min at 1100 °C under a constant Ar flow of 50 sccm.
