# Supplementary material for: β-Ga2O3 Nanostructures: Chemical Vapor Deposition Growth Using Thermally Dewetted Au Nanoparticles as Catalyst and Characterization
Source: Nanomaterials (Basel). 2022 Jul 28;12(15):2589. doi: 10.3390/nano12152589 (PMC9370184; doi:10.3390/nano12152589)
Supplement: Supplementary file 1 [file nanomaterials-12-02589-s001.zip › nanomaterials-1801618-supplementary.pdf]

Supplementary Materials

β-Ga_2O_3 Nanostructures: Chemical Vapor Deposition Growth Using Thermally Dewetted Au Nanoparticles as Catalyst and Characterization

Asha Yadav [1], Bo Fu [2], Stephanie Nicole Bonvicini [1], Linh Quy Ly [1], Zhitai Jia [2] and Yujun Shi [1,*]

[1] Department of Chemistry, University of Calgary, Calgary, AB T2N 1N4, Canada; ashayadav@eee.sastra.edu (A.Y.); snbonvic@ucalgary.ca (S.N.B.); linh.ly2@ucalgary.ca (L.Q.L.)
[2] State Key Laboratory of Crystal Materials, Shandong University, Jinan 250100, China; 201712452@mail.sdu.edu.cn (B.F.); z.jia@sdu.edu.cn (Z.J.)
* Correspondence: shiy@ucalgary.ca; Tel.: +1-403-2108674

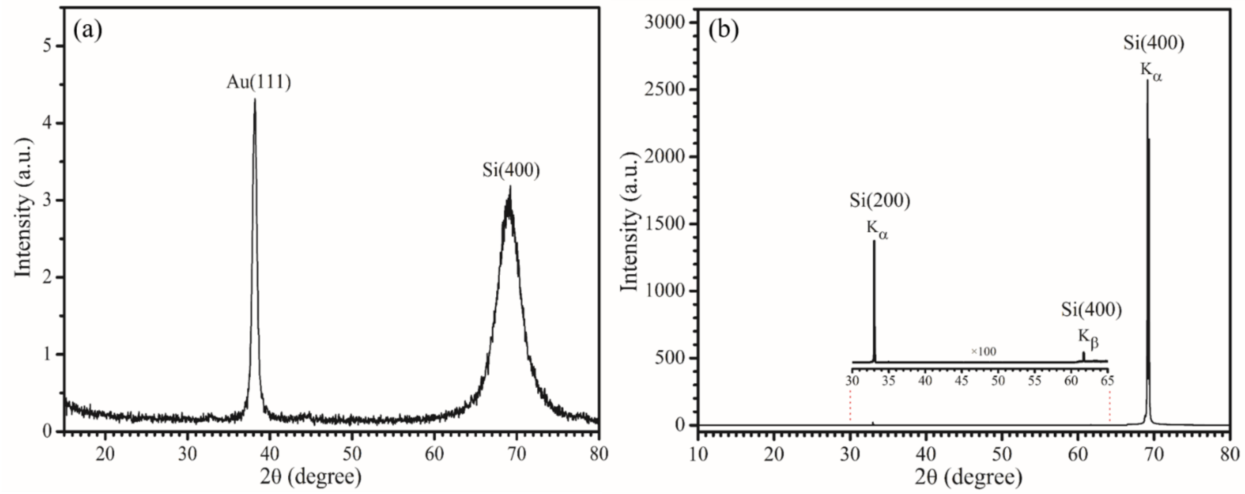

Figure S1. XRD patterns of (**a**) Au nanoparticles from thermal dewetting of a 2.9 nm thick Au thin film on a Si substrate, and (**b**) a blank Si (100) substrate. Peak assignments are based on ICDD No. 04-0784 for Au and No. 80-0018 for Si.

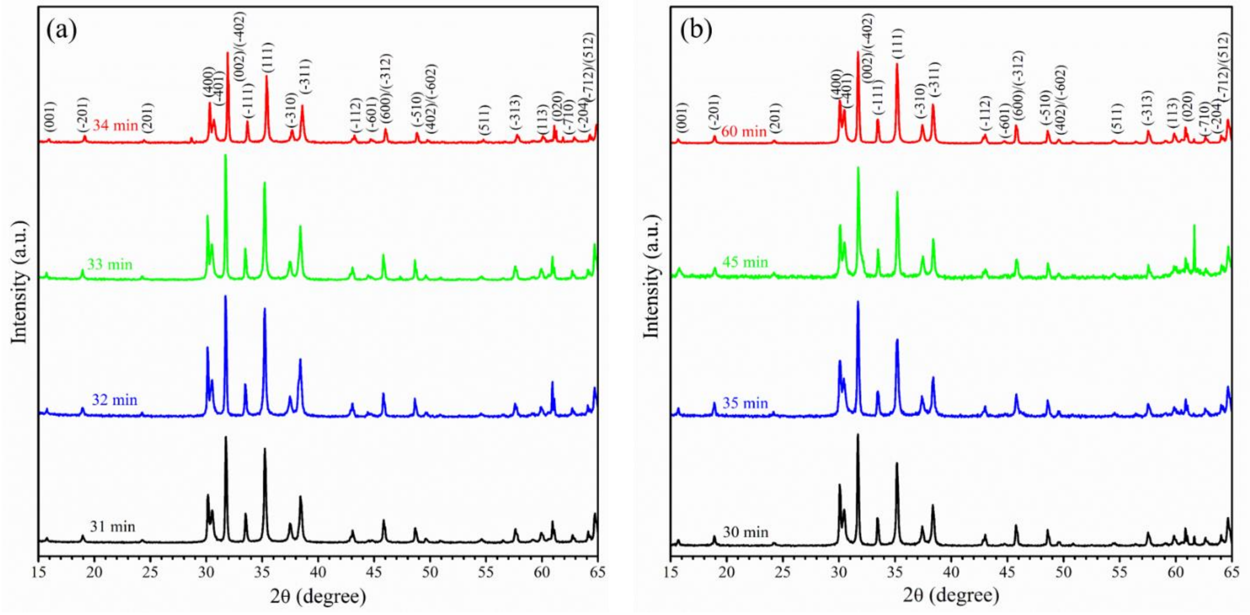

Figure S2. XRD patterns of *β*-Ga_2O_3 nanostructures grown for various times of (**a**) 31−34 min at 1000 °C and (**b**) 30−60 min at 1100 °C under a constant Ar flow of 50 sccm.

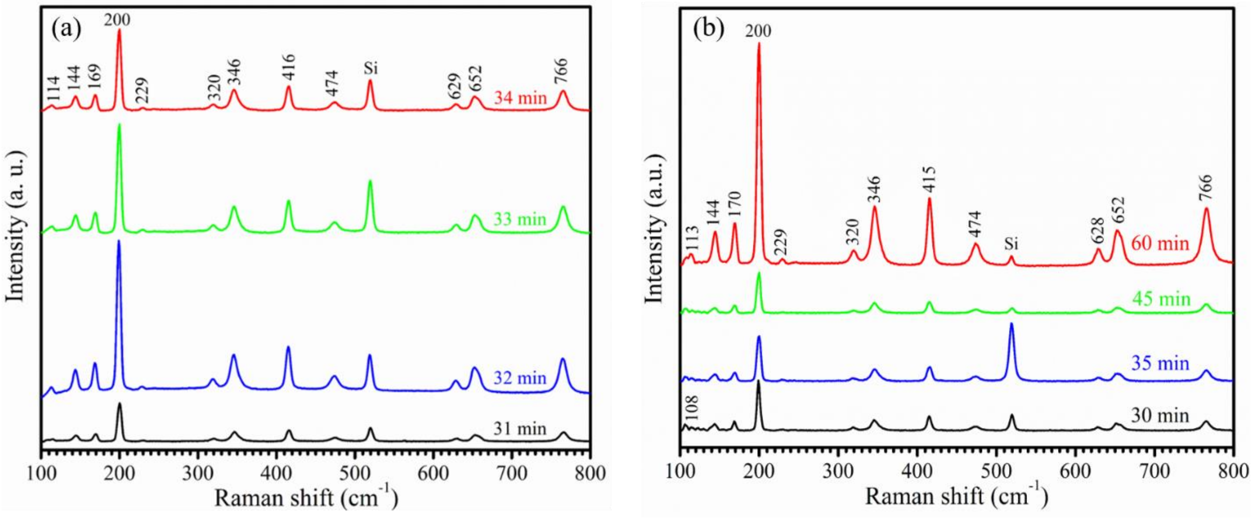

Figure S3. Room-temperature Raman spectra of *β*-Ga_2O_3 nanostructures grown for various times of (**a**) 31−34 min at 1000 °C and (**b**) 30−60 min at 1100 °C under a constant Ar flow of 50 sccm.
